# Supplementary material for: Impact of predictor measurement heterogeneity across settings on the performance of prediction models: A measurement error perspective
Source: Stat Med. 2019 May 31;38(18):3444–59. doi: 10.1002/sim.8183 (PMC6619392; doi:10.1002/sim.8183)
Supplement: Supplementary file 1 — SIM_8183‐Supp‐0001‐SIM‐18‐0491_SuppFileText.pdf [file SIM-38-3444-s001.pdf]

APPENDIX 1

In this appendix, the general effects of measurement heterogeneity on external predictive performance are illustrated in large sample simulations ($N = 1,000,000$).

| Simulation design

We examined the predictive performance of a single-predictor binary logistic regression model. The data were generated from

$$\text{logit}(Y) = \log(8)X,$$
$$\text{where } X \sim \mathcal{N}(0, 0.5),$$

and where X reflects the true (often unobserved) underlying value of the predictor. The dataset contained two measurements of the predictor x, which were recreated under the general measurement error model (Equation 1). The first measurement, denoted w_D, was used to derive the logistic regression model and corresponded to the random error model (Equation 2). The other measurement, w_V, was used to validate the model and corresponded to various measurement structures under the general measurement error model. This validation procedure implies that the model is validated in its original sample, hence, in absence of all other impacts on model transportability. The `val.prob` function from the rms package in R was used to compute the simulation outcome measures and to generate the calibration plots,[25] where we edited the legend format settings in the plot to improve readability.

| Simulation results

In line with expectations, the predictive performance at validation corresponded perfectly to the predictive performance at derivation when the predictor was measured consistently over settings. The impact on predictive performance when measurements were heterogeneous is described below.

| Random measurement heterogeneity

When the measurement at validation, in w_V, was less precise than at derivation, in w_D, i.e. when $\sigma^2_{\epsilon(D)} < \sigma^2_{\epsilon(V)}$, the c-statistic decreased from 0.71 at derivation to 0.63 at validation and the Brier score increased from 0.22 to 0.26, indicating a loss in discriminatory power and accuracy. Furthermore, the calibration slope was 0.37, similar to statistical overfitting (Figure 5b). When the measurement w_V was more precise than w_D, i.e. when $\sigma^2_{\epsilon(D)} > \sigma^2_{\epsilon(V)}$, the c-statistic increased from 0.71 to 0.81, and the Brier score decreased from 0.22 to 0.20. However, the improved c-statistic and Brier score were accompanied by a calibration slope of $b = 2.42$, similar to statistical underfitting (Figure 5d). Calibration-in-the-large was not affected by random measurement heterogeneity.

| Systematic measurement heterogeneity

Additive systematic measurement heterogeneity, i.e. $\psi_D \neq \psi_V$, resulted in systematic overestimation of the outcome, which is reflected in the negative value for calibration-in-the-large coefficient, -0.22 (Figure 6c). Changes in ψ had no apparent effect on the calibration slope, c-statistic, and Brier score. Multiplicative systematic measurement heterogeneity

at validation, in w_V, i.e. $\theta_V \neq 1$, in combination with random measurement error led to a calibration slope $b < 1$. The impact on the c-statistic and the Brier score was in the direction of association between x and w. When this association was relatively weak, e.g. when $\theta_V = 0.5$, the c-statistic decreased from 0.71 to 0.63 and the Brier score increased from 0.22 to 0.24 (Figure 7b). When the association between x and w_V was relatively strong, e.g. when $\theta_V = 2.0$, the c-statistic improved from 0.71 to 0.77 and the Brier score improved from 0.22 to 0.19 (Figure 7d).

| Differential measurement heterogeneity

All forms of differential measurement of cases and non-cases led to miscalibration at external validation. For example, when measurement of cases was less precise at validation, in w_V, i.e. $\sigma^2_{\epsilon 1(V)} > \sigma^2_{\epsilon 0(V)}$, the calibration slope at validation was 0.54. The c-statistic decreased from 0.71 to 0.66, the Brier score increased from 0.22 to 0.24 (Figure 8a). In case of systematic differential measurement of cases and non-cases, when the association between x and w in cases was weaker in w_V, i.e. $\theta_{1D} > \theta_{1V}$, the c-statistic decreased from 0.71 to 0.68, the Brier score increased from 0.22 to 0.23, and the calibration slope was 0.89 (Figure 8c).

Inverse effects on predictive performance were found when cases and non-cases were measured differentially at derivation, in w_D. When measurement of cases was less precise at derivation, i.e. $\sigma^2_{\epsilon 1(D)} > \sigma^2_{\epsilon 0(D)}$, the c-statistic increased from 0.66 to 0.71, the Brier score decreased from 0.23 to 0.22, and the calibration slope at validation was 1.84 (Figure 9b). When the association between x and w in cases was weaker at derivation, in w_D, i.e. $\theta_{1D} < \theta_{1V}$, the c-statistic improved from 0.68 to 0.71, the Brier score improved from 0.23 to 0.22, and the calibration slope was 1.12 (Figure 9c).
