# Supplementary material for: Impact of predictor measurement heterogeneity across settings on the performance of prediction models: A measurement error perspective
Source: Stat Med. 2019 May 31;38(18):3444–59. doi: 10.1002/sim.8183 (PMC6619392; doi:10.1002/sim.8183)
Supplement: Supplementary file 2 — SIM_8183‐Supp‐0002‐SIM‐18‐0491_SuppFileFig.pdf [file SIM-38-3444-s002.pdf]

SUPPLEMENTARY FIGURE 1

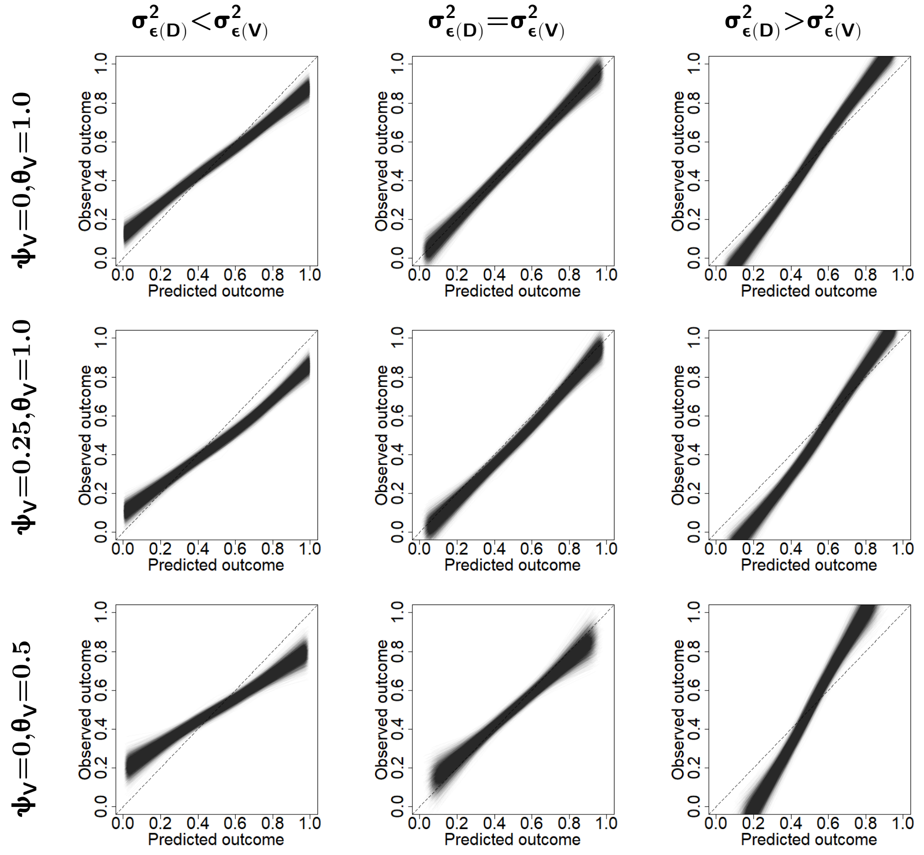

Lowess calibration curves are overlaid for 10,000 resamplings for 9 scenarios of predictor measurement heterogeneity in the two-predictor model in which both predictors are measured heterogeneously. The figure titles indicate the parameters of the general measurement error model (Equation 1) to which the predictor measurements at validation correspond.
