# Supplementary material for: Impact of predictor measurement heterogeneity across settings on the performance of prediction models: A measurement error perspective
Source: Stat Med. 2019 May 31;38(18):3444–59. doi: 10.1002/sim.8183 (PMC6619392; doi:10.1002/sim.8183)
Supplement: Supplementary file 3 — SIM_8183‐Supp‐0002‐SIM‐18‐0491_Fig5.pdf [file SIM-38-3444-s003.pdf]

Random measurement heterogeneity

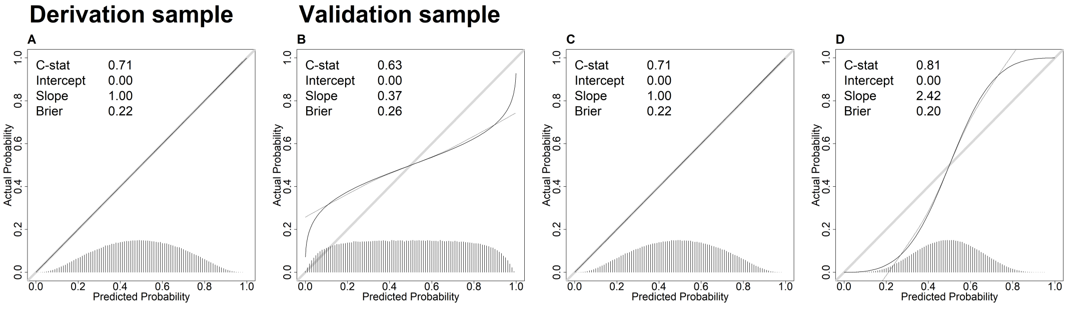

FIGURE 5 Predictive performance of a single-predictor binary logistic regression model. The predictor measurement structure corresponds to:

A. $W_D = X + \epsilon_D$, where $X \sim \mathcal{N}(0, 0.5)$ and $\epsilon_D \sim \mathcal{N}(0, 0.5)$.
B. $W_V = X + \epsilon_V$, where $X \sim \mathcal{N}(0, 0.5)$ and $\epsilon_V \sim \mathcal{N}(0, 2.0)$. Measurements are less precise at validation, i.e. $\sigma^2_{\epsilon(D)} < \sigma^2_{\epsilon(V)}$.
C. $W_V = X + \epsilon_V$, where $X \sim \mathcal{N}(0, 0.5)$ and $\epsilon_V \sim \mathcal{N}(0, 0.5)$. Measurements consistent across settings, i.e. $\sigma^2_{\epsilon(D)} = \sigma^2_{\epsilon(V)}$.
D. $W_V = X$, where $X \sim \mathcal{N}(0, 0.5)$. Measurements are more precise at validation, i.e. $\sigma^2_{\epsilon(D)} > \sigma^2_{\epsilon(V)}$.
