# Supplementary material for: Impact of predictor measurement heterogeneity across settings on the performance of prediction models: A measurement error perspective
Source: Stat Med. 2019 May 31;38(18):3444–59. doi: 10.1002/sim.8183 (PMC6619392; doi:10.1002/sim.8183)
Supplement: Supplementary file 4 — SIM_8183‐Supp‐0004‐SIM‐18‐0491_Fig6.pdf [file SIM-38-3444-s004.pdf]

Additive systematic measurement heterogeneity

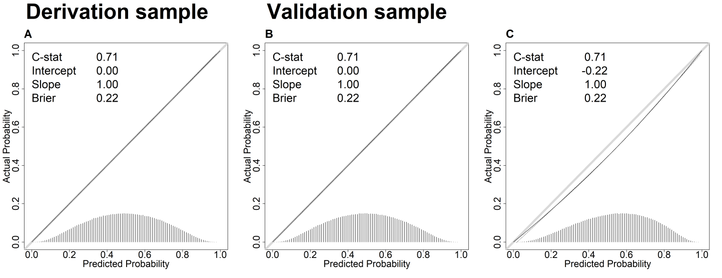

FIGURE 6 Predictive performance of a single-predictor binary logistic regression model. The degree of additive error in the validation predictor measurement W_V varies, while the degree of random error is consistent across settings, i.e. while $\sigma^2_{\epsilon(D)} = \sigma^2_{\epsilon(V)}$. The predictor measurement structure corresponds to:

A. $W_D = X + \epsilon_D$, where $X \sim \mathcal{N}(0, 0.5)$ and $\epsilon_D \sim \mathcal{N}(0, 0.5)$.
B. $W_V = \psi_V + X + \epsilon_V$, where $\psi_V = 0$, $X \sim \mathcal{N}(0, 0.5)$ and $\epsilon_V \sim \mathcal{N}(0, 0.5)$. Measurements are equal across settings.
C. $W_V = \psi_V + X + \epsilon_V$, where $\psi_V = 0.25$, $X \sim \mathcal{N}(0, 0.5)$ and $\epsilon_V \sim \mathcal{N}(0, 0.5)$. Measurements are shifted from X by a constant.
