# Supplementary material for: Impact of predictor measurement heterogeneity across settings on the performance of prediction models: A measurement error perspective
Source: Stat Med. 2019 May 31;38(18):3444–59. doi: 10.1002/sim.8183 (PMC6619392; doi:10.1002/sim.8183)
Supplement: Supplementary file 6 — SIM_8183‐Supp‐0006‐SIM‐18‐0491_Fig8.pdf [file SIM-38-3444-s006.pdf]

Differential measurement heterogeneity at validation

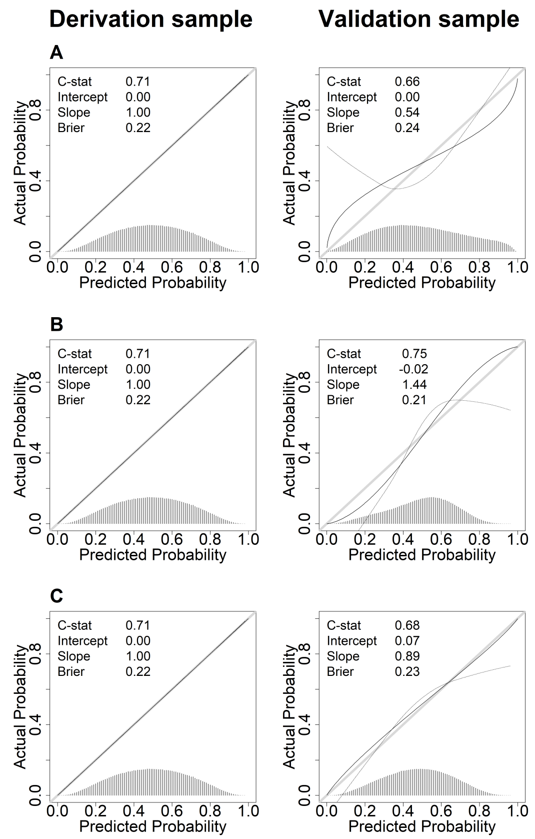

FIGURE 8 Predictive performance of a single-predictor binary logistic regression model. In all three scenarios, $\psi_{D\{0,1\}}$ and $\psi_{V\{0,1\}}$ equal 0, the default value for $\theta_{D\{0,1\}}$ and $\theta_{V\{0,1\}}$ is 1.0, and the default value for $\sigma^2_{\epsilon(D\{0,1\})}$ and $\sigma^2_{\epsilon(V\{0,1\})}$ is 0.5. Otherwise, the predictor measurement structure for the cases at validation (specified by θ_{V1} and $\sigma^2_{\epsilon(V1)}$) corresponds to:

A. $W_V = \theta_V X + \epsilon_V$, where $X \sim \mathcal{N}(0, 0.5)$ and $\epsilon_{V1} \sim \mathcal{N}(0, 2.0)$. Measurements of cases are less precise at validation.
B. $W_V = \theta_V X + \epsilon_V$, where $X \sim \mathcal{N}(0, 0.5)$ and $\epsilon_{V1} \sim \mathcal{N}(0, 0)$. Measurements of cases are more precise at validation.
C. $W_V = \theta_V X + \epsilon_V$, where $\theta_{V1} = 0.5$, $X \sim \mathcal{N}(0, 0.5)$ and $\epsilon_V \sim \mathcal{N}(0, 0.5)$. Associations between X and W in cases are weaker at validation.
