# Supplementary material for: Impact of predictor measurement heterogeneity across settings on the performance of prediction models: A measurement error perspective
Source: Stat Med. 2019 May 31;38(18):3444–59. doi: 10.1002/sim.8183 (PMC6619392; doi:10.1002/sim.8183)
Supplement: Supplementary file 7 — SIM_8183‐Supp‐0007‐SIM‐18‐0491_Fig9.pdf [file SIM-38-3444-s007.pdf]

Differential measurement heterogeneity at derivation

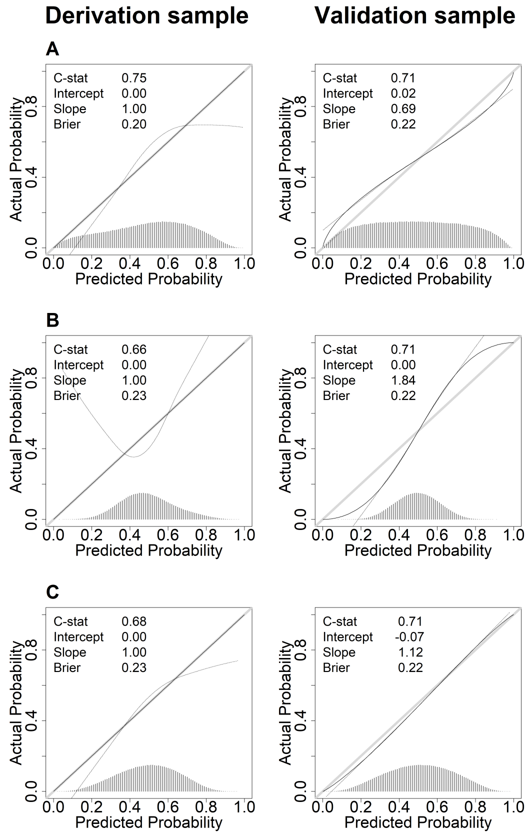

FIGURE 9 Predictive performance of a single-predictor binary logistic regression model. In all three scenarios, $\psi_{D\{0,1\}}$ and $\psi_{V\{0,1\}}$ equal 0, the default value for $\theta_{D\{0,1\}}$ and $\theta_{V\{0,1\}}$ is 1.0, and the default value for $\sigma^2_{\epsilon(D\{0,1\})}$ and $\sigma^2_{\epsilon(V\{0,1\})}$ is 0.5. Otherwise, the predictor measurement structure for the cases at derivation (specified by θ_{D1} and $\sigma^2_{\epsilon(D1)}$) corresponds to:

A. $W_D = \theta_D X + \epsilon_D$,	where $X \sim \mathcal{N}(0, 0.5)$ and $\epsilon_{D1} \sim \mathcal{N}(0, 0)$.	Measurements of cases are more precise at derivation.
B. $W_D = \theta_D X + \epsilon_D$,	where $X \sim \mathcal{N}(0, 0.5)$ and $\epsilon_{D1} \sim \mathcal{N}(0, 2.0)$.	Measurements of cases are less precise at derivation.
C. $W_D = \theta_D X + \epsilon_D$,	where $\theta_{D1} = 0.5$, $X \sim \mathcal{N}(0, 0.5)$ and $\epsilon_D \sim \mathcal{N}(0, 0.5)$.	Associations between X and W in cases are weaker at derivation.
